# Healthcare professionals’ perspectives on artificial intelligence in patient care: a systematic review of hindering and facilitating factors on different levels

**DOI:** 10.1186/s12913-025-12664-2

**Published:** 2025-05-01

**Authors:** Dennis Henzler, Sebastian Schmidt, Ayca Koçar, Sophie Herdegen, Georg L. Lindinger, Menno T. Maris, Marieke A. R. Bak, Dick L. Willems, Hanno L. Tan, Michael Lauerer, Eckhard Nagel, Gerhard Hindricks, Nikolaos Dagres, Magdalena J. Konopka

**Affiliations:** 1 Institute of Management for Medicine and Health Sciences, University of Bayreuth, Prieserstr. 2, Bayreuth, 95444 Germany; 2 Department of Ethics, Law and Humanities, Amsterdam UMC, De Boelelaan 1089a, Amsterdam, 1081 HV The Netherlands; 3 Department of Clinical and Experimental Cardiology, Heart Center, Amsterdam UMC, Meibergdreef 9, Amsterdam, 1105 AZ The Netherlands; 4 Netherlands Heart Institute, Moreelsepark 1, Utrecht, 3511 EP The Netherlands; 5 German Heart Center of the Charité-University Medicine Berlin, Augustenburger Pl. 1, Berlin, 13353 Germany; 6 Department of Epidemiology, Maastricht University, Peter Debyeplein 1, 6229 HA Maastricht, The Netherlands

**Keywords:** Artificial intelligence, Barriers, Facilitators, Healthcare professionals, Perspectives

## Abstract

**Background:**

Artificial intelligence (AI) applications present opportunities to enhance the diagnosis, prognosis, and treatment of various diseases. To successfully integrate and utilize AI in healthcare, it is crucial to understand the perspectives of healthcare professionals and to address challenges they associate with AI adoption at an early stage. Therefore, the aim of this review is to provide a comprehensive overview of empirical studies that explore healthcare professionals’ perspectives on AI in healthcare.

**Methods:**

The review was conducted according to the Preferred Reporting Items for Systematic Reviews and Meta-Analyses framework. The databases MEDLINE, PsycINFO, and Web of Science were searched in the timeline of 2017 to 2024 using terms related to ‘healthcare professionals’, ‘artificial intelligence’, and ‘perspectives’. Eligible were peer-reviewed articles that employed quantitative, qualitative, or mixed-methods approaches. Extracted facilitating and hindering factors were analysed according to the dimensions of the socio-ecological model.

**Results:**

Our search yielded 4,499 articles published up to February 2024. After title abstract screening, 150 full-texts were assessed for eligibility, and 72 studies were ultimately included in our synthesis. The extracted perspectives on AI were thematically analyzed using the socioecological model in order to identify various levels of influence and to categorize them into facilitating and hindering factors. In total, we identified 49 facilitating and 43 hindering factors across all levels of the socioecological model.

**Conclusions:**

The findings from this review can serve as a foundation for developing guidelines for AI implementation adressing various stakeholders, from healthcare professionals to policymakers. Future research should focus on the empirical adoption of AI applications and, if possible, further examine the hindering factors associated with different types of AI.

**Supplementary Information:**

The online version contains supplementary material available at 10.1186/s12913-025-12664-2.

## Introduction

Artificial intelligence (AI) is increasingly being used to process and interpret large sets of medical data [1]. Despite the ongoing development and testing of AI applications in healthcare, the implementation of medical AI systems in clinical care remains in its early stages [2, 3]. AI technology has broad applications within healthcare, including diagnosis and treatment, promoting patient engagement and adherence, and supporting administrative processes [4]. For example, AI could potentially predict critical diseases or health events before they occur [5] or by assessing the relative risk of disease for individuals, AI could inform preventive measures [6, 7]. Furthermore, AI can also be applied to medical research and drug development (e.g., automated manufacturing), health systems management and planning (e.g., resource allocation), and public health activities (e.g., health promotion, surveillance, and outbreak response) [5, 8, 9].

To realize these potentials, the implementation of AI in healthcare requires an understanding of the perspectives of key stakeholders, including healthcare professionals, patients, health managers, leaders, and regulators, who will use or be affected by this emerging technology [10]. For this purpose, a systematic review of the hindering and facilitating factors affecting the implementation of a technology or program can provide stakeholders with relevant information [11]. This knowledge can help identify potential challenges, mitigate risks, and maximize the benefits associated with medical AI in clinical applications, informing the development of targeted strategies for professionals directly impacted by its implementation.

**Fig. 1:**
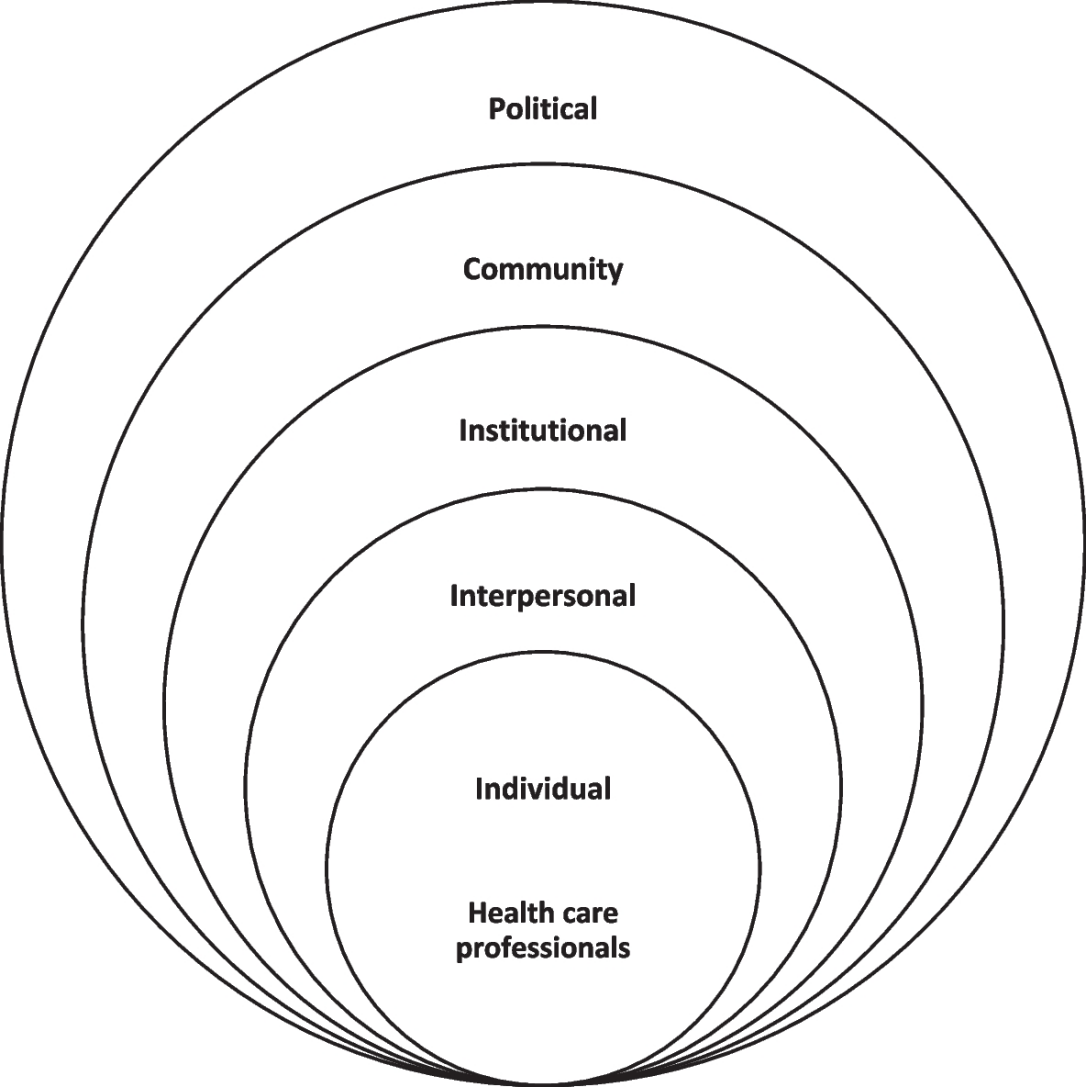
Socio-ecological framework for healthcare professionals’ perspectives on the facilitating and hindering factors to implementing and utilizing AI in healthcare

Several reviews have already examined the perspectives of various stakeholders. For example, a review by Young and colleagues [12] summarized the literature on patient and public attitudes toward AI applications in healthcare. Other reviews have focused on the perspectives of healthcare professionals on topics not directly involving patient care, such as research and drug development [13, 14]. Therefore, this systematic review’s aim is to provide a comprehensive synthesis of available evidence on the factors that hinder or facilitate AI’s role in patient care from the perspective of healthcare professionals. This, in turn, enables a deeper examination of how healthcare professionals perceive AI’s impact on their roles, as well as its implications for their organizations and patients. To better account for the dynamic interaction of facilitating and hindering factors operating at multiple societal levels, we utilize the socio-ecological model (SEM). This model helps identify both individual determinants and contextual influences on healthcare professionals’ behaviour, as well as their ability or inability to implement and utilize AI in practice. In this review, the SEM places healthcare professionals at the center, surrounded by five levels of influence: individual, interpersonal, institutional, community, and policy (see Fig. 1).

This systematic review is based on the main research question: “What are the perceived hindering and facilitating factors for the implementation and use of AI by healthcare professionals that are involved in direct patient care?”. While a recent integrative review by Lambert et al. [15] explored the facilitators and barriers influencing AI acceptance among healthcare professionals, this review adds to this approach in several ways. Lambert et al. presented their results based on the Unified Theory of Acceptance and Use of Technology (UTAUT), which explains a user’s intention to adopt information technology systems. In contrast, the present systematic review places greater emphasis on contextual factors, such as specific fields of medicine, adapting the SEM as its theoretical framework.

Additionally, Lambert et al. primarily included studies on Clinical Decision Support Systems (CDSS) and limited their review to hospital settings. To account for the differences among AI types, this review employs the typology proposed by Davenport and Kalakota, which categorizes AI systems into machine learning, natural language processing models, rule-based expert systems, and robotic process automation [4]. Finally, the search conducted by Lambert et al. in June 2022 was followed by a significant increase in AI-related studies in medicine, which could be included in this review.

The paper is structured as follows: The introduction presents an overview of previous research on the topic and highlights the review’s unique contributions to the literature. The methods section outlines the methodological approach for searching, screening, extracting and synthesizing data from the included studies. Descriptive results are presented based on the Preferred Reporting Items for Systematic Reviews and Meta-Analyses (PRISMA) flow diagram and key study characteristics. Data analysis and synthesis results are categorized in facilitating and hindering factors, structured according to the dimensions of the SEM. In the discussion, findings are interpreted in relation to the healthcare professionals’ perspectives on the anticipated changes of AI on their professional role, their organizations, and patients. Also, the study’s limitations are addressed. The conclusion summarizes key insights and provides an outlook on future research.

## Methods

### Eligibility criteria

Our research methodology followed all required elements of the 2020 PRISMA checklist for systematic reviews (see Additional file 1) [16], except for a quality appraisal of included studies. Given that our aim was on describing the variety of perspectives among healthcare professionals rather than synthesizing findings across studies to determine the size of an effect or compare study results, no formal assessment of study quality was performed. Eligible studies were peer reviewed articles investigating healthcare professionals’ perspectives on clinical AI, either hypothetical or already implemented in patient care. Only studies in English and German were included due to the linguistic expertise of the research team. Primary empirical studies that examined perspectives on AI of healthcare professionals working in direct patient care (e.g., physicians, nurses, medical-technical staff, etc.) were included. The same applies for quantitative, qualitative and mixed-methods studies. Articles were excluded if not based on primary research or if the reports focused only on general technological development, not AI specifically. Moreover, articles investigating healthcare professionals with no direct patient contact or substantial experience such as students or laboratory workers as well as mixed populations (patients and professionals together, if not reporting stratified results) were excluded. Finally, we excluded reviews, comments, case reports, letters, editorials and other forms of grey literature (e.g., theses, conference proceedings) since only empirical studies to review healthcare professionals’ perspectives regarding the (hypothetical) use of AI in healthcare were of interest.

### Study selection and data extraction

MEDLINE via PubMed, as well as the PsychInfo and Web of Science databases, were searched on February 14, 2024. Search terms were clustered in three seach concepts: artificial intelligence, healthcare professionals, and perspectives. The search was restricted to original articles published in 2017 or later since following the first FDA approval of AI/ML medical technologies in the year 2016, with three approvals at the end of the year 2017 [17], there has been exponential growth in the application of AI in healthcare [18]. Finally, the filter 'NOT review' was applied in all searches to exclude reviews from the search results. The detailed search strategies are listed in the appendix (see Additional file 2). Identified studies from the databases were extracted to Endnote (Clarivate Analytics, Version 21.3) and automatically screened for duplicates. Titles and abstracts of the retrieved reports were initially read by two reviewers (SH, MK). Consequently, two independent reviewers (SH and DH) screened the full texts of articles that seemed eligible for inclusion. Disagreement between SH and DH was solved by discussion and in consultation with MK. Data extraction was performed independently by three reviewers (DH, MK, SeS) using MS Excel. In accordance with common practice for quantitative survey studies, factors with a level of agreement ≥ 70% were extracted and included in the present review [19]. This means, items that received a level of agreement less than 70% of the total study participants were excluded. In qualitative studies all factors were extracted. Risk of bias and certainty assessments were not applicable [20].

### Synthesis of results

**Fig. 2:**
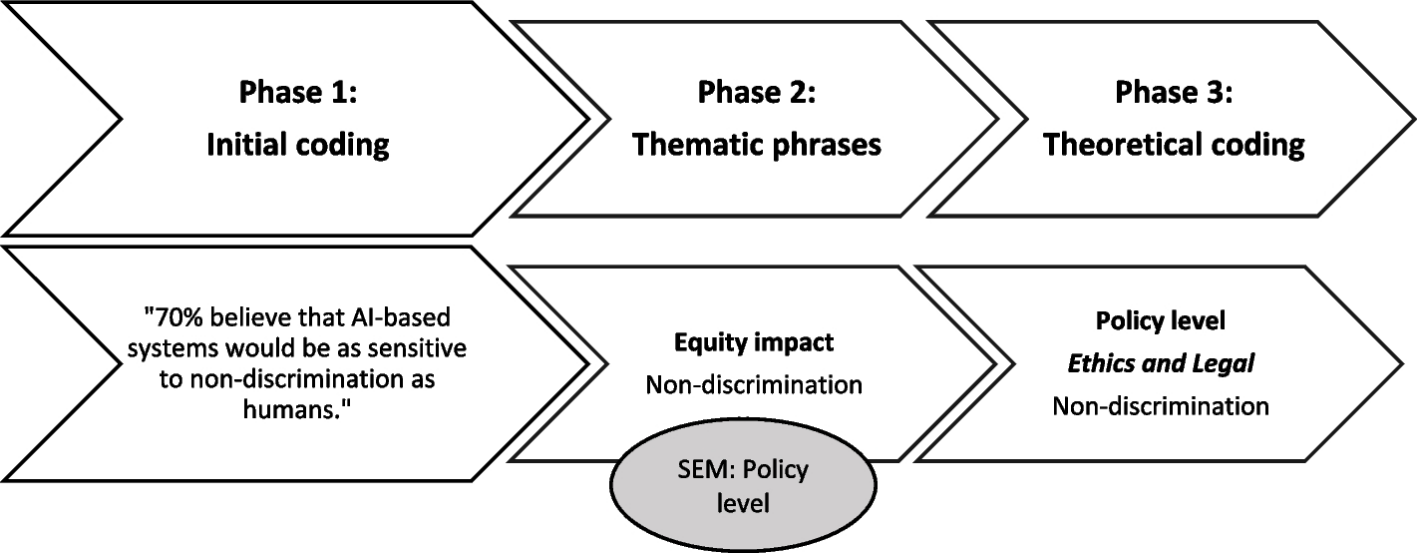
Illustration of the analysis process of a facilitating factor

For the synthesis of results, the extracted facilitating and hindering factors were thematically analyzed and categorized into themes [21]. A facilitating factor is defined as one that positively influences healthcare professionals' perceptions of AI in patient care, while a hindering factor refers to elements that negatively impact their persception of AI in the workplace. The coding strategy consisted of three stages: i) initial coding: remaining open to all possible themes indicated by initial readings of the articles, ii) focused coding: categorizing the data inductively based on thematic similarity, and finally iii) theoretical coding: integrating thematic categories [22]. Figure 2 illustrates the analysis process.

### Socio-ecological model

The facilitating and hindering factors are categorized and analyzed using the socio-ecological model as a reference framework developed by Urie Bronfenbrenner [23]. The SEM framework is widely applicable to various factors affecting health systems and practices [24–27] making it well-suited to assess the complex influences on AI implementation across different contexts.

The thematic phrases were placed into one or more contextual levels of influence across the SEM. Firstly, the *individual* level describes behaviors as well as knowledge, attitudes, and perspectives. Individuals' knowledge, perspectives, and experiences are key in influencing their response in any given situation. In implementing AI into practice, the responsibility for delivering patient care extends across healthcare professionals, each with various backgrounds, training, and motivation for their role. Secondly, *interpersonal* factors describe relationships and networks developed by individuals with their coworkers or professionals in clinical settings. Thirdly, *institutional* factors include operational elements and aspects of the physical environment that contribute to how successfully they can implement AI, including technical aspects of AI. Fourthly, the *community* level covers the relationship of healthcare professionals with other organizations and media, which can affect their ability to implement AI. Finally, the *policy* level includes local, state, and federal policies and laws that regulate or support actions and practices in medicine. The results are presented in tabular form according to SEM dimensions in the result section, inspired by the work of Ma et al., [25].

## Results

### Study selection

**Fig. 3:**
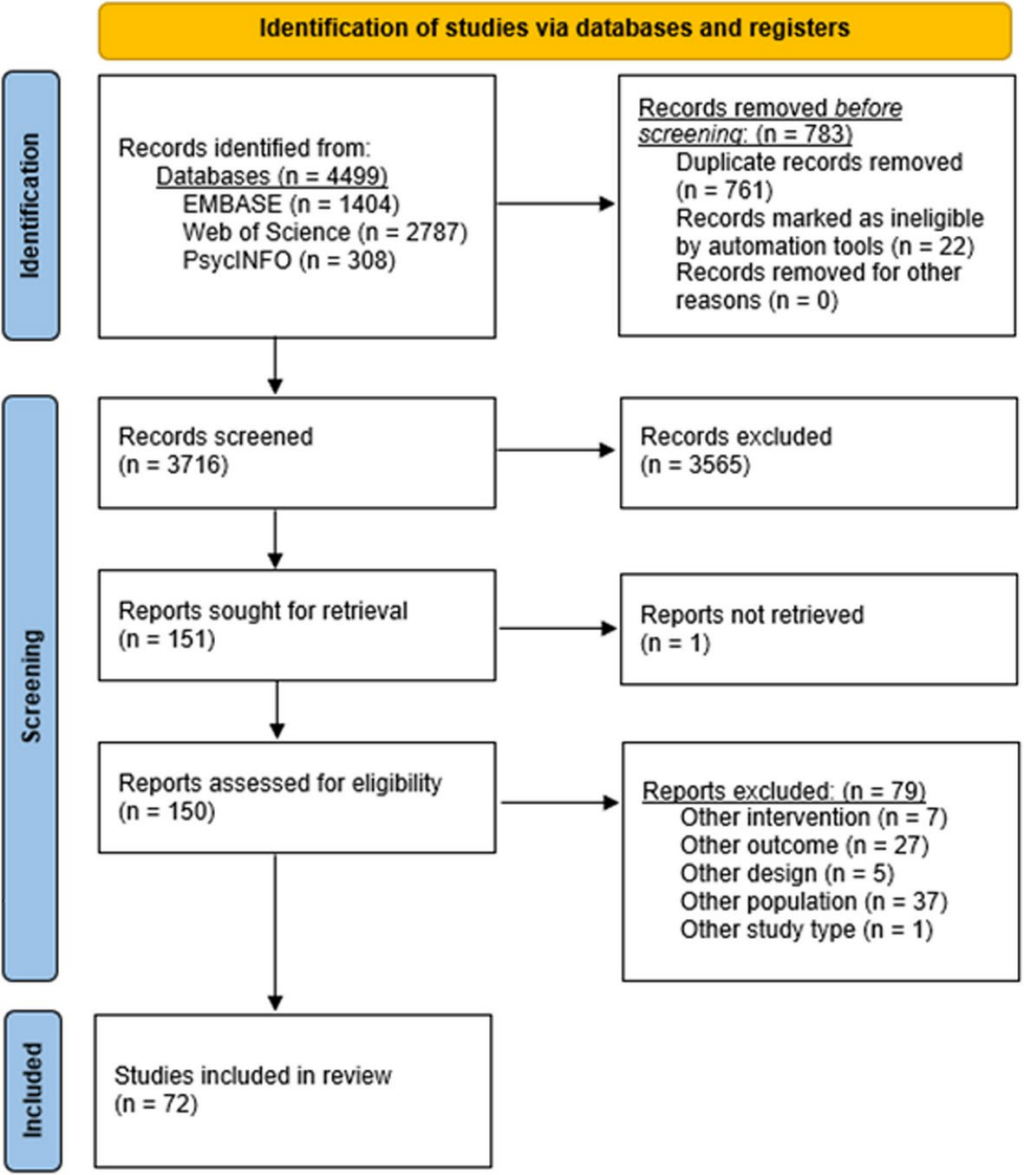
PRISMA flow diagram of studies in the review

The literature search identified 4,499 records. Of the initial dataset, 761 duplicates were removed as well as 22 non‐English or non-German studies. Consequently, 3,716 articles were screened based on title and abstract thereby excluding another 3,565 records. Next, 151 articles were read for detailed evaluation of which one article [28] was not retrievable even after contacting the corresponding author. Of the remaining 150 studies, 72 were eligible for inclusion. Figure 3 depicts the flow of study selection. The reasons for study exclusion were other intervention (*n* = 7), outcome (*n* = 27), study design (*n* = 5), population (*n* = 37), or study type (*n* = 1) and study retraction (*n* = 1). The reasons for exclusion are summarized in the appendix (see Additional file 3).

### Study characteristics

In the appendix the key characteristics of the included studies containing the facilitating and hindering factors identified are depicted (see Additional file 4). The analysis of 72 articles revealed diverse perspectives from healthcare professionals across various regions. The studies were roughly distributed geographically as follows: 33 were conducted in Europe, 16 in Asia, 12 in America, four in Africa, and three in Australia, while four studies spanned multiple countries. These studies collectively investigated the perspectives of 15,325 healthcare professionals, including lab technicians, midwives, nurses, physical therapists, among others, and physicians from different medical specialities. The healthcare professionals were predominantly from the specialities of radiology (*n* = 20), primary care (*n* = 4), oncology (*n* = 4), orthodontics (*n* = 3), sexual and reproductive health (*n* = 3), (neuro)surgery (*n* = 3), ophthalmology (*n* = 3), pathology (*n* = 2), physical therapy (*n* = 2), pharmacy (*n* = 2), dermatology (*n* = 2). Additionally, other specialties included psychiatry (*n* = 1), otolaryngology (*n* = 1), emergency medicine (*n* = 1), gastroenterology (*n* = 1), haemodialysis (*n* = 1), paediatricians (*n* = 1), nephrology (*n* = 1), venereology (*n* = 1), and anaesthesiology (*n* = 1), with several studies not specifying the healthcare professionals' field of medicine. Methodologically, the research comprised 43 quantitative studies, 17 qualitative studies, and 12 employing mixed-methods designs.

**Fig. 4:**
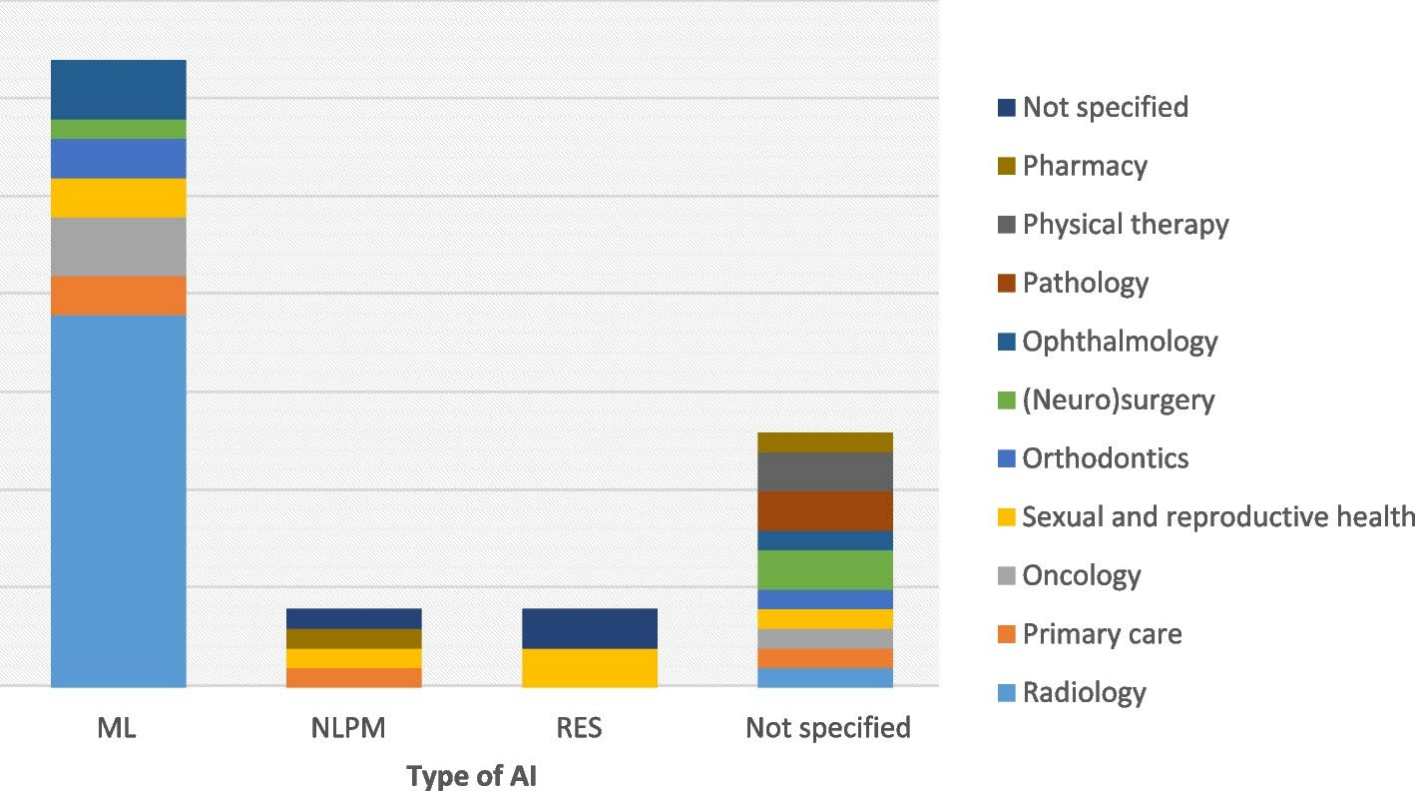
Field of medicine (reported n > 1) stratified by “type of AI”. AI = artificial intelligence, ML = machine learning, NLPM = natural language processing model, RES = rule-based expert systems

The included studies showed that the actual implementation of AI systems in healthcare settings is still a work in progress. Most of them (*n* = 62) evaluated hypothetical deployment or scenario-based implementation of AI tools. The majority of studies were either machine/deep learning (30/72) or AI in general not specifying the type of AI (29/72). A handful of studies could be clearly assigned as “natural language processing models” [29–32] or “rule-based expert systems” [33–36]. Figure 4 depicts the different types of AI stratified by the field of medicine. It was not possible to differentiate between hindering and facilitating factors by the type of AI due to the limited number of studies that could be clearly assigned to the AI type.

### Facilitating factors

Fifty-eight of the 72 studies included in this review described facilitating factors of healthcare professionals for the use of AI in healthcare settings (Table 1). The facilitating factors are discussed according to individual, interpersonal, institutional, community and policy levels. Overall, 49 different facilitating factors were identified across multiple levels of the social-ecological model. Most facilitating factors were identified at the individual level (*n* = 14) and institutional level (*n* = 19), while fewer factors were found at the interpersonal level (*n* = 3) and community level (*n* = 4).

**Table 1:** Facilitating factors

Individual level	Interpersonal level	Institutional level	Community level	Policy
***Knowledge about AI*** • Familiarity [34, 37, 38] • Time to trial AI in practice [39] • Education and training [39] • Conferences and networks [40] ***Attitude towards profession*** • No fear of job loss [41, 42] • Improves self-assurance [43] • Educational function [38] • Retaining decision control [36, 39, 44, 45] ***Management of own knowledge*** • Time efficiency (e.g. quick access, summarizing information) [29, 30, 46–49] • Handling of big data in real-time [50, 51] ***Working with AI*** • Decrease time on repetitive tasks (medical, non-medical) [30, 40, 46, 52–55] • Task delegation to AI [47] • More time and focus on patient and/or critical tasks [45, 56] ***Useability*** • Ease of use technology (user-friendliniess), useability [36, 38, 39]	***Implication of relationships to patients*** • Communication with patients (e.g. chatbot, multilingual) [30, 32] ***Relationship with coworkers*** • Teamwork and Coordination [30, 35, 57] • Collaboration across teams (e.g. AI team, physicians, IT) [36]	***Medical decision-making in clinical setting*** • Complex cases, connecting multiple sources of information (e.g. drug interaction, potential contraindications) [29, 34, 54, 58] • Personalized recommendation [30, 32] • Decision support [45, 59] • Second opinion, treatment accuracy [60–62] • Improved sensitivity/specifity, reduce missed diagnoses [31, 33, 38, 44, 46, 52, 55, 60–75] • Risk assessment for appropriate patient pathway [33, 76] ***Workflow/processes in clinical setting*** • Efficiency [38, 41, 44, 55, 56, 62, 67, 68, 77–80] • Standardized reporting [69] • Time efficiency [52, 66] • Process optimization (e.g. triage of patients) [46, 57, 81] • Reduce workload [82–84] • Real time alert of hazards and complications [57, 72] ***Quality of care ***[80] • Patient safety (reduce medical mistakes) [32, 46, 49, 72] • Improve medical standards [85] • Improvement of clinical outcomes [33, 54, 55, 61, 79, 84] • Consistency of care (no exhaustion, no hunger, no emotion) [46, 61, 66] • Timeliness of care [44] • Scientific objectivity [69] • Health improvement, no side effects [36, 44, 52]	***Research community*** • Endorsed by leaders, academic societies, trusted experts [38, 52] • Transparency in the development process [63] • Explainability and verifiability [63] • Evidence-based technology, large RCTs to support the validity, reliability and effectiveness [36, 38, 39, 42, 58]	***Support healthcare system*** • Relieve workforce crisis [40, 44] • Potential for cost efficient, time efficiency [44] • Improve population health indicators [79] • Access to healthcare [32, 59, 60, 71] • Remote support for patients [34] ***Ethics and Legal*** • Uniformity and evidence-based in diagnosis [54, 65] • Non discrimination of patients [44, 86] • Clear legal framework (e.g. data protection, liability) [37, 38, 40]

#### Individual level

Five studies reported on the facilitating factors at the indivudual level that encourage greater knowledge about AI by healthcare professionals, including being familiar with AI [34, 37, 38], having time to trial the AI application in their practice [39] and receiving education and training programs [39] as well as conferences and scientific networks [40] as opportunities to learn about them. Eight studies reported on facilitating factors that relate to the professionals’ attitude towards their profession, such as as reducing their fear of job superflousness [41, 42]. Healthcare professionals would prefer to see themselves as retaining the overall control of the treatment process of their patients and that AI is used as a decision-support and not a replacement [36, 39, 44, 45], while also acknowledging that AI can also serve as a tool for educational purposes [38] and can lead to more self-assurance in their decisions [43]. Ten studies describe factors relating to AI’s impact on their clinical tasks, such as an overall decreased time spent on repetitive medical and non-medical tasks [30, 40, 46, 52–55] and the possibility to delegate other tasks to AI [47], which in turn could allow them to focus more time and focus on direct interaction with their patients or other perceived critical tasks [45, 56]. Furthermore, the professionals believe that AI would increase their efficiency in managing medical information and knowledge, by being granted quick access to vast amounts of knowledge with the ability to scan and summarize relevant information effectively with the help of AI [29, 30, 46–49] or being able to handle big-data records in real-time [50, 51]. Other facilitating factors include the ease of use and user-friendliness of AI technology, ensuring that it is accessible and usable for healthcare professionals [36, 38, 39].

#### Interpersonal level

Five studies reported on facilitating factors at the interpersonal level, highlighting positive implications for relationships with patients and interactions with coworkers. Positive developments in their interaction with patients are mainly seen through enhanced communication [30, 32] with the ability to provide immediate responses support with chatbots or using AI tools for translating and communicating with patients who speak different languages. In terms of relationships with coworkers, AI facilitates better teamwork and coordination among healthcare professionals [30, 35, 57] but require also to foster collaboration across different professionals teams, such as AI specialists, physicians, and IT professionals [36]. For example, one paper suggests that “ChatGPT could enhance collaboration among healthcare teams by facilitating communication, sharing knowledge and insights, and coordinating care across multiple providers” [30].

#### Institutional level

Facilitating factors were linked to medical decision-making within clinical settings, efficient workflows and processes in clinical settings, and general improvements in the quality of care. In terms of medical decision-making in clinical settings, most studies reported on benefits relating to a better diagnostic quality in either improved sensitivity and specificity or reducing the likelihood of missed diagnoses [31, 33, 38, 44, 46, 52, 55, 60–75]. More specified uses were seen in treating complex cases by being able to connect multiple sources of information (e.g. drug interactions) [29, 34, 54, 58], supporting the risk assessment for the determination of appropriate patient pathways [33, 76], as well as, providing personalized treatment recommendations such as providing tailored recommendations and treatment plans based on the patient’s medical history, preferences, and lifestyle factors [30, 32], a general decision support [45, 59] or offering second opinions to improve treatment accuracy [60–62].

Regarding the perceived benefits of using AI for the workflows and processes in institutional settings, many studies reported an expected increase in overall efficiency [38, 41, 44, 45, 55, 56, 62, 67, 77–80] as a facilitating factor, a reduction in overall workload [82–84] or improved time management [52, 66]. Further facilitating factors were seen as general optimization of clinical processes [46, 57, 81], which would also allow for a betterment in standardized reporting [69] and monitoring alert of hazards and complications in real time [42, 57], contributing to safer clinical environments.

The main quality of care benefits from AI integration is seen as an overall improvement in clinical outcomes [33, 54, 55, 79, 80, 84] by increasing patient safety [32, 46, 49, 72] and enhancing medical standards [85]. Furthermore, AI contributes to better clinical outcomes and ensures consistency of care by mitigating human factors like exhaustion [46, 61, 66]. It also might aid in achieving adequate timeliness of care for the patient [44] and grounds therapeutic decisions in scientific objectivity [69].

#### Community level

Seven studies reported on facilitating factors on community level, that mainly address the research community. Hereby, a facilitating factor is that the outcomes and clinical effectivenes of AI applications should be evidence-based, ideally relying on large randomized controlled trials (RCTs) to support claims about its effectiveness and safety [36, 38, 39, 42, 58], while the development process itself should be transparent [63] and the AI system used should be explainable and verifiable [63]. Furthermore, endorsements from leaders within their fields, academic societies, and trusted experts lend credibility and acceptance to AI technologies [38, 52]. No further facilitating factors for other communities could be identified.

#### Policy level

Facilitating factors at the policy level included healthcare system support as well as ethical and legal considerations. Support for the healthcare system includes relieving the currently experienced workforce crisis by healthcare professionals [40, 44]. AI applications would offer system-wide potential to enhance care delivery by improving cost and time efficiency [44]. Overall system-wide benefits for patients are seen in an improved access to healthcare services [32, 59, 60, 71], for instance by establishing remote support for patients [34], and by improving population health indicators by supporting measures of disease prevention and health promotion [79]. Ethical facilitators are seen insofar that AI systems would not discriminate against patients [44, 86] while also providing equal care to all which is evidence-based, ensuring that all patients receive fair and equitable treatment [54, 65]. For example, Turkish specialists in emergency departments believe that AI could be as sensitive to issues of non-discrimination as humans, if not more so [86]. Finally, clear legal frameworks are essential [37, 38, 40], covering aspects such as data protection and liability, to safeguard patient information and provide clarity on the responsibilities and accountabilities associated with AI use in healthcare.

### Hindering factors

Fifty-five of the 72 studies included in this review described hindering factors of healthcare professionals for the use of AI in a healthcare settings from which 43 different hindering factors were extracted (Table 2). Most hindering factors were identified at the individual (*n* = 11) and institutional levels (*n* = 13), while fewer factors were attributed to the interpersonal level (*n* = 6).

**Table 2:** Hindering factors

Individual level	Interpersonal level	Institutional level	Community level	Policy level
***Knowledge about AI*** • Lack of education programs [37, 56, 87, 88] • Lack of knowledge [32, 42, 52, 55, 59, 69, 71, 76, 80, 83, 87, 89, 90] • Time constraint for education [37, 39, 40] • Age of healthcare professionals [39] ***Attitude towards profession*** • Fear of job replacement [32, 38, 48, 91] • Fear of dependency, overreliance, loss of competency [30, 58, 61, 69, 73, 76] • Job requirements [58] ***Working with AI*** • Conflict of opinion [92] • Information used by AI is not up to date [30] • Increased workload [31, 32, 38, 61] ***Other*** • Overloaded by technology [31]	***Implication of relationships to patients*** • Impact on doctor-patient relationship [31, 36, 45, 46, 52, 76] • Communication with patient [29, 82] • Lack of human touch [30, 32, 46, 47, 52, 77, 78, 82, 83] • AI disclosure to patient [30] • Patient compliance depends on useability [43]	***Medical decision making in clinical setting*** • Reliability (AI not up to date, trust issues) [30, 69, 76, 93] • Clinical errors [30, 31, 38, 40, 46, 52, 55, 81, 84, 85] • Decreased sensitivity/specifitity [61] • Inability to account for patient diversity, complex or controversial cases, context [29–35, 48, 52, 55, 61, 76, 83] • Limitation of programming scope [32, 83] • Inadequacy in specific contexts [46] ***Organizational readiness*** • Lack of responsible personnel (Chief Officer or Office) [87] • Lack of organisational support [37] • Lack of funding [42] • Compatibility of treatment methods and digital systems [62] ***Organizational costs*** • Implementation costs [37, 39, 54, 75, 94] • Education and training [83] • Development and Acquisition [83, 91]	***Healthcare organizations*** • Dehumanization of healthcare [29, 47] • Commercial interests [37, 38, 45] • inappropriate use by insurance companies [69] ***Research and development*** • Lack of transparency in research, development and validation [33, 36, 38] • Bias in training data (e.g. color of skin) [38] • Explainability and interpretability of AI [37, 39]	***Healthcare system issues*** • Divestment of healthcare to large technology companies [65] • Lack of adequate reimbursement models [31] ***Equity issues*** • Health inequalities [32, 61] • Inequitable healthcare quality due to AI use [38] ***Legal issues*** • Unclear responsibility [41, 65, 86] • Liability and accountability [30, 31, 61, 65, 70, 76, 80] • Security and privacy (data) [30, 38, 45, 46, 52, 65, 76, 91] • Lack of regulatory policies [45, 56, 64]

#### Individual level

At the individual level, several hindering factors were identified, concerning the knowledge of AI, attitudes towards the profession, and working with AI, as well as other aspects such as technology overload. Knowledge of healthcare professionals about AI is limited [32, 42, 52, 55, 59, 69, 71, 76, 80, 83, 87, 89, 90] and hindered by a lack of suitable education programs [37, 56, 87, 88], time constraints of healthcare professionals to make use of education programs [37, 39, 40], and advanced age of healthcare professionals, which may affect their ability to adapt to new technologies [39]. Eleven studies reported hindering factors related to the anticipated negative impact on their roles and profession and are influenced by a fear of job replacement [32, 38, 48, 91] and a fear of dependency on AI as well as overreliance on technology, and concerns about loss of competency [30, 58, 61, 69, 73, 76]. Additionally, the evolving job requirements to integrate AI into practice may be an additional burden [58]. Working with AI presents challenges such as conflicts of opinion between healthcare professionals and AI systems [92], the use of outdated information by AI [30], and worrying about increasing the overall workload [31, 32, 38, 61].

#### Interpersonal level

Nineteen studies reported on the hindering factors at the interpersonal level that concerned the relationship between healthcare professionals and patients. AI's lack of empathy and human touch, considered essential in healthcare, can diminish this relationship [30, 32, 46, 47, 52, 77, 78, 82, 83]. Additionally, communication with patients may be hindered if AI takes over the primary conversational role [29, 82]. Further hindering factors can be seen in the necessary disclosure of AI use to patients, as patients may be wary or mistrustful of AI involvement in their care [30]. Hence, patients should have the right to know if they are interacting with an AI or a human teleconsultant, which can be a burden in terms of transparency, privacy and trust. Furthermore, patient compliance may depend on the user friendliness of AI systems, with less user-friendly technologies potentially leading to reduced adherence to medical advice and treatment plans [43].

#### Institutional level

At the institutional level, various factors impede the integration of AI in clinical decision-making, organizational readiness, and cost management. In clinical settings, reliability issues can arise from outdated AI systems, leading to trust concerns [30, 69, 76, 93]. The main hindering factor for the implementation of AI is seen by the healthcare professionals in the possibility of clinical errors that can lead to patient harm [30, 31, 38, 40, 46, 52, 55, 81, 84, 85]. This risk is mainly attributed to AI’s inability to adequately consider patient diversity and complex cases [29–35, 48, 52, 55, 61, 76, 83], which may prevent AI from functioning effectively in specific contexts [46] or expose limitations in its programming scope [32, 83].

Organizational readiness mainly refers to the structural requirements to successfully implement AI applications in healthcare organizations. For instance, the lack of responsible personnel [87], insufficient funding [42], overall lack of organizational support [37], such as from the organization’s leadership, and compatibility issues between AI systems and existing clinical treatment methods as well as the in-use digital system [62] are seen as key hindering factors. Furthermore, the high costs of implementation [39, 54, 75, 83, 91], necessary education and training [83], as well as development and acquisition of AI technologies [83, 91] are financial barriers.

#### Community level

At the community level, hindering factors were associated with how services are provided at healthcare organizations and how AI systems are developed within research and development communities. In healthcare organizations, the dehumanization of healthcare [29, 47] is seen as hindering factor. Furthermore, commercial interests of private companies that develop AI systems might be untrustworthy [37, 38, 45], including a fear that AI systems might be used inappropriately by health insurance companies [69]. For the organizations that perform research and development of AI systems, a lack of transparency in the development and validation of AI systems [33, 36, 38], bias in training data (such as issues related to skin color) [38], and concerns about the explainability and interpretability of AI systems [37, 39] are seen as obstacles to effective implementation and AI technologies.

#### Policy level

At the policy level, hindering factors are categorized into broader healthcare system issues and more specific ethical and legal issues related to the use of AI and the consequences thereof. Concerning the implementation of AI in the healthcare system, healthcare professionals worry about divesting large amounts of resources in healthcare away from the provision of healthcare services to large technology companies [65], while also fearing that the system lacks an adequate reimbursement model for financing AI systems in healthcare organizations [31]. Broader equity issues are seen insofar that the use of AI can increase health inequalities in the population [32, 61], including creating different classes of healthcare quality in the organizations that use AI and in those that do not, given AI improves healthcare quality [38]. Healthcare professionals’ concerns about legal issues include unclear responsibilities between AI and physicians [41, 65, 86], questions of liability and accountability in the face of clinical errors [30, 31, 61, 65, 70, 76, 80] concerns about data security and privacy of patient data [30, 38, 45, 46, 52, 65, 76, 91], and the lack of comprehensive regulatory policies governing AI in healthcare [45, 56, 64].

## Discussion

This review demonstrates that categorizing perspectives of healthcare professionals according to the characteristics of the SEM helps to understand the hindering and facilitating factors for the use of and attitudes towards AI applications. Furthermore, it allows to identify recommendations for taking action for mitigating barriers or strengthening facilitators at the different SEM levels. A large part of the included studies (*n* = 34) were published from the year 2022 onwards, which demonstrates the need for an upate on the perspectives of healthcare professionals on AI applications, as the emergence of AI tools is continuously rising. Thus, more recent studies could be included in this study than similar reviews up to this point [15]. An initial intention of this review was to highlight differences in facilitating and hindering factors for different types of AI applications. However, no differences could be identified due to the low amount of studies that could be clearly categorized into the different types of AI according to Davenport and Kalakota, [4].

To contextualize the findings, the most frequently identified hindering and facilitating factors of the different SEM dimensions are shortly summarized, discussed and recommendations derived thematically according to the healthcare professionals perspectives about how AI applications might impact (i) healthcare professionals, (ii) patient care settings, (iii) patient outcomes. A reflection of limitations concludes the discussion section.

### Healthcare professionals (“How does AI impact me as a healthcare professional?”)

The potential positive effects that can be accomplished by the use of AI applications for work-related tasks are seen as one of the most prominent facilitating factors by healthcare professionals. Increased efficiency is mostly reported in general terms, but more concrete examples state that working with AI can improve individual workflows and time management by providing quick access to (summarized) relevant information, but can also decrease the time spent on administrative or repetitive tasks, such as scheduling appointments, reminders to patients or prescription refills [30]. Tasks such as summarizing information, performing administrative tasks or scheduling appointments can be performed by NLPM, such as ChatGPT [95, 96]. In this review, only four studies focused on NLPM applications in medicine [29–32]. Hereby, perceived hindering factors were a reduction of human touch in medical care, communication challenges wih patients and open questions about the liability and accountability as well as security and privacy issues of patient data, which are further discussed in the following sections. These hindering factors all relate to tasks of direct patient care, whereas some perceived and expected efficiency gains as pointed out in this review, are not necessarily directly related to medical tasks but also to administrative tasks or the healthcare professionals' own knowledge management.

AI applications that impact the work conditions and workflows for direct patient care also need to account for the fact that many healthcare professionals express a fear or concern of (future) dependency on AI applications, insofar that their own skills and competencies may diminish as a consequence of relying too much on the technology. These concerns are not necessarily AI specific and have been voiced at earlier technological developments in medicine, such as with the implementation of the electronic health record [97]. It is difficult to ascertain if AI implementation will lead to a loss of clinical skills. However, to compensate the use of AI will demand the development of new skills of healthcare professionals, e.g. information management skills, and strengthen the skills that AI most likely cannot substitute for such as communication capabilities and empathy [98].

Also, a lack of general knowledge about AI or adequate educational programs, or not being able to participate in educational programs are considered hindering factors in either having or acquiring the necessary knowledge about AI applications. One way to mitigate this factor is to develop medical curricula and educational methods to train future doctors the fundamentals of AI, its effective use in practice, and AI-supported healthcare delivery [99]. However, as the knowledge gaps persist in the current generation of active healthcare professionals it might be necessary to increase further training opportunities in the workplace or as part of a professional continuing education programs.

### Healthcare organisations (“How does AI impact my healthcare organisation?”)

A variety of facilitating and hindering factors are seen by healthcare professionals in the impact AI might have on the patient care setting. Here, the prospect of increased efficiency in the healthcare organization is seen as facilitating factor on the institutional level that might also relieve tensions in the overall workforce crisis on the healthcare system stemming from the increased lack of healthcare personnel. Also, abilities of AI applications, such as improving diagnostic accuracy or connecting multiples sources of information to detect drug interactions or potential contraindications, which can lead to personalized recommendations and offer second opinions, showcase the expected impact of AI that healthcare professionals believe it will have on the patient care setting. However, to realize the potential benefits that AI might bring to patient care settings, it is important that the organizations are prepared for the implementation of the new technologies and can adequately support its staff in adapting to it.

As indicated in this review, barriers at the organisational level can be structural, such as lack of technical infrastructure, initial funding or long-term reimbursement models, but also because there is a lack of responsible personnel or department dedicated to the implementation of AI applications in healthcare organizations or policy level. For instance, in Germany regulations and governance issues have delayed the nationwide implementation of rather basic healthcare technologies, such as the electronic health record [100]. For the implementation of new technology, studies highlight the relevance of individuals (“healthcare leaders”) in the adaptation process in healthcare organizations [101]. For instance, the inclusion of clinical personnel as advocates (“champions”) for a new technology is a positive factor for implementation of new technologies in healthcare organizations [102]. Thus, identifying the right persons to drive those changes in the organizations might be an important factor for overcoming a variety of hindering factors. This notion resonates further with the finding of this review that the endorsement of trusted experts or health leaders constitutes a facilitating factor for using AI by healthcare professionals [38, 52].

To address hindering factors surrounding the patient care setting, governments and healthcare organizations should prioritize investment in digital health infrastructure. Furthermore, specialized departments should focus on AI strategy and implementation, managed by persons in leadership roles, who are responsible for overseeing the integration of AI into clinical and operational processes.

### Patient outcomes (“How does AI impact my patients?”)

Another topic for which both facilitating and hindering factors were identified relate to the healthcare professionals' perspectives about the impact of AI applications on their patients’ health and well-being. Most of the facilitating factors are seen as an impact on the quality of care, such as overall improvements in clinical outcomes, patient safety as well as providing high-quality care that is consistent and not impacted by a healthcare professional's state of mind or circumstances like fatigue. This is for the most part seen on an individual patient level, but some positive attitude is also expressed for the improvement of overall population health [79] as well as access to healthcare services overall.

On the other hand, potential harm to patients caused by AI and the lack of human touch in healthcare provision are the most frequent hindering factors stated by healthcare professionals. Especially, healthcare professionals fear that AI applications might not be able to account for patient diversity, complex cases, and contextual social nuances that may limit its clinical effectiveness as well as moral appropriateness in specific situations. There was uncertainty whether AI could incorporate important individual aspects gained through the physician–patient relationship [52], make complicated ethical judgments [29] or handle “corner cases” that are unexpected or unique [76]. This also includes worries that the data sets on which AI-models are being trained on are not representative of the population to which they are applied [61].

These hindering factors seem to reveal a contradiction to the beliefs of healthcare professionals that AI can improve the quality of care by facilitating more personalized care. An advantage of using the SEM is its ability to reveal that by looking at one or both hindering and facilitating factors within the same dimensions can often address the same issue or uncover additional insights. It is seen as a key facilitating factor that AI has the potential to handle more complex cases by integrating and connecting multiple sources of information. This highlights that while AI is seen as capable of managing patient diversity and even reducing medical complexity, there are other aspects of diversity that AI is perceived to handle less effectively. Categorizing these factors according to the SEM levels thus provides a more comprehensive picture of facilitators and barriers of each dimension.

Furthermore, healthcare professionals are concerned that even with human oversight clinical errors might result from outdated or poorly programmed AI systems and pose risks to patient safety either by incorrect diagnoses or leading to inappropriate treatments. These factors might be further exacerbated by the lack of clarity about the division of responsibilities in the clincal care process and the accountability of the outcomes thereof. These concerns are substantiated in the desire of many healthcare professionals to precondition the use of AI applications on clinical trials, with ideally large RCTs supporting the validity of its results [36, 38, 39, 42, 58]. Furthermore, the generalizability of such studies and tested AI applications need to be considered carefully. As AI or machine learning applications’ outcomes in medicine can rely to a large amount on the data they receive for training, differences in genomic or environmental factors may influence disease patterns and the presentation of diseases. Thus, the development of AI applications should ideally be based on data from different ethnic groups and regionally tested to validate their efficacy [103]. Furthermore, RCTs of medical AIs may not always examine medical-biological mechanisms but rather organizational or procedural pathways in how diagnostic and therapeutic practices are changed. Thus, researchers should ensure that patient outcomes are stable across time, patient characteristics, and across clinicians of different specializations or levels of experience [104].

### Limitations

Some limitations should be considered when interpreting the findings of the present review. First, the included studies show that the actual implementation of AI systems in healthcare settings and clinics is still a work in progress. Even though in most quantitative studies participants would state that they had either knowledge or use-experience of AI applications, this could not be verified or aggregated in a meaningful way. Thus, most of the findings of the healthcare professionals' perspectives are considered as not having experience with clinical AI. As mentioned elsewhere, there is a need for studies investigating AI applications in real-world clinical settings [105]. Second, a majority of the studies that were identified in this review are assigned to the disciplines of radiology (AI-based radiology image analysis) or to unspecified general medicine (broadly defined as AI implementation in medicine). This may limit the generalizability of the findings to other medical fields, even though the perspectives of healthcare professionals from a total of 20 different medical disciplines could be included. Also, no differences in facilitating and hindering factors could be identified looking at the type of AI. This is due to the fact that only four studies involving NLPM or RES could be identified, respectively. For further research it is critical to differentiate the type of AI as concretely as possible so that differences according to AI type can be more easily attributed. Also, a reviewer pointed out to us, that the applied typology from Davenport and Kalakota [4] for AI in medicine might be slightly dated given the dynamism of the field. Yet, the typology remains widely cited in the scientific literature, establishing it as a relevant framework.^1^ Nonetheless, future researchers could benefit from applying more recent frameworks that reflect the latest advancements in the field.

A possible limitation of this review is the inclusion of only three databases and the exclusion of grey literature, such as dissertations, reports, or conference proceedings, which could introduce publication bias. While including these sources might have broadened the scope of our review, the decision to focus on peer-reviewed primary research articles was made to ensure a high standard of methodological quality. Additionally, our review was restricted to studies published in English and German, which may have led to the omission of relevant research in other languages. However, only 22 out of 3,738 studies were excluded based on language before the title and abstract screening process. Although we cannot rule out the possibility that additional relevant studies exist, given the inclusion of 72 studies and the low proportion of excluded non-English/German studies, it is unlikely that that these limitations substantially impacted the comprehensiveness of the review or altered our conclusions. Also, no quality assessment of the included studies was performed for this review, which may have led to the inclusion of lower quality studies.

Finally, while the use of the SEM constitutes a helpful framework to break down the complexity of perspectives towards AI into different hindering and facilitating factors, some limitations became apparent. First, choosing the levels to which a certain identified theme belongs was not always clear. For instance, expected “efficiency gains” through the use of AI technology could be identified at the individual, institutional and policy level. This difficulty was approached by looking closer at the intention or aim of the given statement, thus, clarifying if efficiency realisations were meant to be for the healthcare professionals to become more productive personally, the overall workflow and processes at the patient care setting or if overall efficiency realisations could be accomplished at the healthcare systems perspective (policy level). When working with the SEM it is also difficult to gauge how factors at each level might influence each other [25, 106, 107]. For instance, it might be of interest how a “lack of human touch” in the healthcare process impacts the doctor-patient-relationship and, consequently, patient care outcomes. Especially for practical implementation purposes identifiying these influences and interdependencies might be important where context is an essential factor. However, the current review aims at mapping these factors only. Future research might look into these questions in more detail.

## Conclusion

In conclusion, this systematic review explored healthcare professionals’ perspectives on the factors that facilitate and hinder the use of AI in patient care. Overall, we found that healthcare professionals generally hold a positive view to adopt AI in healthcare and expect various positive impacts for the provision of health services to their patients. However, various hindering factors must be addressed and tailored to meet the specific needs of healthcare professionals and other stakeholders. The review also revealed that the implementation of clinical AI involves complex factors across different socio-ecological dimensions. Therefore, it is crucial to take action at multiple levels to ensure the successful integration of AI in healthcare. Our findings can serve as a foundation for developing guidance for AI implementation across various stakeholders, from healthcare professionals to policymakers. Further research should focus on the perspectives of AI currently in use in healthcare settings and explore the differences in facilitating and hindering factors among various types of AI. It is critical that primary studies clearly specify the type of AI being examined. Furthermore, qualitative studies are especially important, as they can provide new insights from healthcare professionals who already have experience with AI in their workplaces.

## Supplementary Information

Additional file 1. Preferred Reporting Items for Systematic Reviews and Meta-Analyses (PRISMA) 2020 checklist with references to the main manuscript.

Additional file 2. Search strategy for the databases MEDLINE via PubMed, PsychInfo, and Web of Science.

Additional file 3. Excluded studies with reason for exclusion.

Additional file 4. Characteristics and results of included studies.

## Data Availability

All relevant data is provided within the manuscript or supplementary information files.

## Abbreviations

AI: Artificial Intelligence
CDSS: Clinical Decision Support System
FDA: Food and Drug Administration
ML: Machine Learning
NLPM: Natural Language Processing Model
PRISMA: Preferred Reporting Items for Systematic Reviews and Meta-Analyses
RCT: Randomized Controlled Trial
RES: Rule-based Expert System
SEM: Socio-Ecological Model
UTAUT: Unified Theory of Acceptance and Use of Technology

## Footnotes

1 Number of citations are taken from GoogleScholar: 3,478 citations in total; 2024: 1,220; 2025: 162 (last checked on 18.02.2025).
