# Supplementary material for: Healthcare professionals’ perspectives on artificial intelligence in patient care: a systematic review of hindering and facilitating factors on different levels
Source: BMC Health Serv Res. 2025 May 1;25:633. doi: 10.1186/s12913-025-12664-2 (PMC12046968; doi:10.1186/s12913-025-12664-2)
Supplement: Supplementary file 2 — Additional file 2. Search strategy for the databases MEDLINE via PubMed, PsychInfo, and Web of Science. [file 12913_2025_12664_MOESM2_ESM.pdf]

Additional file 2 Search strategy

Database MEDLINE via PubMed:

**ID**	**Search string**	**Results (n)**
1	(((((((((Doctor[Title/Abstract]) OR (Physician[Title/Abstract])) OR (Health Personnel[Title/Abstract])) OR (Professional[Title/Abstract])) OR (Provider[Title/Abstract])) OR ("Physicians"[Mesh])) OR ("Health Personnel"[Mesh])) AND ((((((artificial intelligence[Title/Abstract]) OR (Machine learning[Title/Abstract])) OR (Deep learning[Title/Abstract])) OR ("Artificial Intelligence"[Mesh])) OR ("Machine Learning"[Mesh])) OR ("Deep Learning"[Mesh]))) AND ((((((((((Opinion[Title/Abstract]) OR (Perspective[Title/Abstract])) OR (View*[Title/Abstract])) OR (Experience[Title/Abstract])) OR (Preference[Title/Abstract])) OR (attitude[Title/Abstract])) OR (perception[Title/Abstract])) OR (perceive[Title/Abstract])) OR (Belie*[Title/Abstract])) OR ("Attitude"[Mesh]))) NOT (Review[Publication Type]) Publication Date from 2017/1/1 - 2024/2/14	**1,404**

Database Web of Science:

ID	Search string	Results (n)
1	TS=(doctor) Editions: WOS.SCI	108657
2	TS=(physician) Editions: WOS.SCI	344017
3	TS=("health personnel") Editions: WOS.SCI	4732
4	TS=(professional) Editions: WOS.SCI	297505
5	TS=(provider) Editions: WOS.SCI	190461
6	#2 OR #3 OR #4 OR #5 OR #1 Editions: WOS.SCI	837926
7	TS=("artificial intelligence") Editions: WOS.SCI	91576
8	TS=("machine learning") Editions: WOS.SCI	228347
9	TS=("deep learning") Editions: WOS.SCI	150766
10	#7 OR #8 OR #9 Editions: WOS.SCI	406450
11	TS=(opinion) Editions: WOS.SCI	127686
12	TS=(perspective) Editions: WOS.SCI	663197
13	TS=(view*) Editions: WOS.SCI	800919
14	TS=(experience) Editions: WOS.SCI	1510898
15	TS=(preference) Editions: WOS.SCI	321112
16	TS=(attitude) Editions: WOS.SCI	218351
17	TS=(perception) Editions: WOS.SCI	423158
18	TS=(perceive) Editions: WOS.SCI	283998
19	TS=(belie*) Editions: WOS.SCI	395274
20	#11 OR #12 OR #13 OR #14 OR #15 OR #16 OR #17 OR #18 OR #19 Editions: WOS.SCI	3981613
21	#6 AND #10 AND #20 Editions: WOS.SCI	3388
22	DT=(Review) Editions: WOS.SCI	2400832
23	(#21) NOT DT=(Review) Editions: WOS.SCI	2945
24	#23 Editions: WOS.SCI Timespan: 2017-01-01 to 2024-02-14	**2787**

Database PsycINFO:

ID	Search string	Results (n)
S28	((S16 OR S17 OR S18OR S19 OR S20 OR S21OR S22 OR S23 OR S24OR S25) AND (S8 ANDS15 AND S26)) NOT PZreview PublicationDate: 20170101-20240331	308
S27	(S16 OR S17 OR S18 ORS19 OR S20 OR S21 ORS22 OR S23 OR S24 ORS25) AND (S8 AND S15AND S26)	388
S26	(S16 OR S17 OR S18 ORS19 OR S20 OR S21 ORS22 OR S23 OR S24 ORS25) AND (S8 AND S15AND S26)	1,805,635
S25	S16 OR S17 OR S18 ORS19 OR S20 OR S21 ORS22 OR S23 OR S24 ORS25	93,233
S24	MA attitude	238,180
S23	TI belie* OR AB belie*	49,200
S22	TI perception OR ABperception	351,322
S21	TI attitude OR AB attitude	234,880
S12	MA artificial intelligence	1,824
S11	TI "deep learning" OR AB"deep learning"	3,362
S10	TI "machine learning" ORAB "machine learning"	11,029
S9	TI "artificial intelligence"OR AB "artificialintelligence"	6,966
S8	S1 OR S2 OR S3 OR S4OR S5 OR S6 OR S7	446,121
S7	MA physician	27,200
S6	MA health personnel	30,355
S5	TI provider OR ABprovider	85,317
S4	TI professional OR ABprofessional	278,201
S3	TI "health personnel" ORAB "health personnel"	989
S2	TI physician OR ABphysician	70,303
S1	TI doctor OR AB doctor	31,485
