# Supplementary material for: Healthcare professionals’ perspectives on artificial intelligence in patient care: a systematic review of hindering and facilitating factors on different levels
Source: BMC Health Serv Res. 2025 May 1;25:633. doi: 10.1186/s12913-025-12664-2 (PMC12046968; doi:10.1186/s12913-025-12664-2)
Supplement: Supplementary file 3 — Additional file 3. Excluded studies with reason for exclusion. [file 12913_2025_12664_MOESM3_ESM.pdf]

Additional file 3 Excluded studies with reason for exclusion (n = 80)

Author year	Title	Reason for exclusion
SalehIbrahim 2022	Perception of the Impact of Artificial Intelligence in the Decision-Making Processes of Public Healthcare Professionals	Exclusion reason: Retracted
Barakat 2023	The application of artificial intelligence in diabetic retinopathy screening: a Saudi Arabian perspective	Exclusion reason: Wrong intervention;
Bourla 2018	Psychiatrists' Attitudes Toward Disruptive New Technologies: Mixed-Methods Study	Exclusion reason: Wrong intervention;
Cao 2024	AI triage or manual triage? Exploring medical staffs' preference for AI triage in China	Exclusion reason: Wrong intervention;
Ergin 2022	Artificial intelligence and robot nurses: From nurse managers' perspective: A descriptive cross-sectional study	Exclusion reason: Wrong intervention;
Parikh 2022	Clinician perspectives on machine learning prognostic algorithms in the routine care of patients with cancer: a qualitative study	Exclusion reason: Wrong intervention;
Petitgand 2020	Investigating the Barriers to Physician Adoption of an Artificial Intelligence- Based Decision Support System in Emergency Care: An Interpretative Qualitative Study	Exclusion reason: Wrong intervention;
Wong 2023	EEG based automated seizure detection - A survey of medical professionals	Exclusion reason: Wrong intervention;
European Society of Radiology 2022	Current practical experience with artificial intelligence in clinical radiology: a survey of the European Society of Radiology	Exclusion reason: Wrong outcomes;
Al-Ali 2023	Attitudes Towards Artificial Intelligence Among Dermatologists Working in Saudi Arabia	Exclusion reason: Wrong outcomes;
Alanazi 2023	Clinicians' Views on Using Artificial Intelligence in Healthcare: Opportunities, Challenges, and Beyond	Exclusion reason: Wrong outcomes;
Al-Medfa 2023	Physicians' attitudes and knowledge toward artificial intelligence in medicine: Benefits and drawbacks	Exclusion reason: Wrong outcomes;
Barwise 2023	Using artificial intelligence to promote equitable care for inpatients with language barriers and complex medical needs: clinical stakeholder perspectives	Exclusion reason: Wrong outcomes;
Brkljacic 2019	Impact of artificial intelligence on radiology: a EuroAIM survey among members of the European Society of Radiology	Exclusion reason: Wrong outcomes;
Chawla 2023	Knowledge, Attitude and Perception Regarding Artificial Intelligence in Periodontology: A Questionnaire Study	Exclusion reason: Wrong outcomes;
Chen 2023	Radiology Residents' Perceptions of Artificial Intelligence: Nationwide Cross-Sectional Survey Study	Exclusion reason: Wrong outcomes;
Cobianchi 2023	Surgeons' perspectives on artificial intelligence to support clinical decision-making in trauma and emergency contexts: results from an international survey	Exclusion reason: Wrong outcomes;
DeSimone 2022	Knowledge, attitude, and practice of artificial intelligence in emergency and trauma surgery, the ARIES project: an international web-based survey	Exclusion reason: Wrong outcomes;
Eastwood 2023	Needs and expectations for artificial intelligence in emergency medicine according to Canadian physicians	Exclusion reason: Wrong outcomes;
Elnaggar 2023	Assessment of the Perception and Worries of Saudi Healthcare Providers About the Application of Artificial Intelligence in Saudi Health Facilities	Exclusion reason: Wrong outcomes;

Huisman 2021	An international survey on AI in radiology in 1,041 radiologists and radiology residents part 1: fear of replacement, knowledge, and attitude	Exclusion reason: Wrong outcomes;
Lim 2022	Non-radiologist perception of the use of artificial intelligence (AI) in diagnostic medical imaging reports	Exclusion reason: Wrong outcomes;
Nehme 2023	Performance and attitudes toward real-time computer-aided polyp detection during colonoscopy in a large tertiary referral center in the United States	Exclusion reason: Wrong outcomes;
Polesie 2020	Attitudes Toward Artificial Intelligence Within Dermatopathology: An International Online Survey	Exclusion reason: Wrong outcomes;
Rainey 2022	An insight into the current perceptions of UK radiographers on the future impact of AI on the profession: A cross-sectional survey	Exclusion reason: Wrong outcomes;
Schulz 2023	Modeling the influence of attitudes, trust, and beliefs on endoscopists' acceptance of artificial intelligence applications in medical practice	Exclusion reason: Wrong outcomes;
Shen 2020	Web-based study on Chinese dermatologists' attitudes towards artificial intelligence	Exclusion reason: Wrong outcomes;
Temsah 2023	ChatGPT and the Future of Digital Health: A Study on Healthcare Workers' Perceptions and Expectations	Exclusion reason: Wrong outcomes;
vanderMeijden 2023	Intensive Care Unit Physicians' Perspectives on Artificial Intelligence-Based Clinical Decision Support Tools: Preimplementation Survey Study	Exclusion reason: Wrong outcomes;
Wong 2021	Perceptions of Canadian radiation oncologists, radiation physicists, radiation therapists and radiation trainees about the impact of artificial intelligence in radiation oncology - national survey	Exclusion reason: Wrong outcomes;
York 2023	Clinician and computer: a study on doctors' perceptions of artificial intelligence in skeletal radiography	Exclusion reason: Wrong outcomes;
Zoli 2022	Young Neurosurgeons and Technology: Survey of Young Neurosurgeons Section of Italian Society of Neurosurgery (Società Italiana di Neurochirurgia, SINch)	Exclusion reason: Wrong outcomes;
Samaran 2021	Interest in artificial intelligence for the diagnosis of non-melanoma skin cancer: a survey among French general practitioners	Exclusion reason: Wrong outcomes;
Al-Khaled 2020	Evaluation of physician perspectives of artificial intelligence in ophthalmology: a pilot study	Exclusion reason: Wrong outcomes
Chu 2023	Radiologists' Expectations of Artificial Intelligence in Pancreatic Cancer Imaging: How Good Is Good Enough?	Exclusion reason: Wrong outcomes;
Helenason 2024	Exploring the feasibility of an artificial intelligence based clinical decision support system for cutaneous melanoma detection in primary care - a mixed method study	Exclusion reason: Wrong study design;
Huang 2023	Are physicians ready for precision antibiotic prescribing? A qualitative analysis of the acceptance of artificial intelligence-enabled clinical decision support systems in India and Singapore	Exclusion reason: Wrong study design;
Nitiéma 2023	Artificial Intelligence in Medicine: Text Mining of Health Care Workers' Opinions	Exclusion reason: Wrong study design;
Shamszare 2023	Clinicians' Perceptions of Artificial Intelligence: Focus on Workload, Risk, Trust, Clinical Decision Making, and Clinical Integration	Exclusion reason: Wrong study design;
Chen 2022	Acceptance of clinical artificial intelligence among physicians and medical students: A systematic review with cross-sectional survey	Exclusion reason: Wrong study design;
Eltorai 2020	Thoracic Radiologists' Versus Computer Scientists' Perspectives on the Future of Artificial Intelligence in Radiology	Exclusion reason: Wrong study population;
Abouzeid 2021	Role of Robotics and Artificial Intelligence in Oral Health and Preventive Dentistry - Knowledge, Perception and Attitude of Dentists	Exclusion reason: Wrong study population;

AlZaabi 2023	Are physicians and medical students ready for artificial intelligence applications in healthcare?	Exclusion reason: Wrong study population;
Amann 2023	Expectations and attitudes towards medical artificial intelligence: A qualitative study in the field of stroke	Exclusion reason: Wrong study population;
Ardon 2020	Clinical Laboratory Employees' Attitudes Toward Artificial Intelligence	Exclusion reason: Wrong study population;
Bergquist 2024	Trust and stakeholder perspectives on the implementation of AI tools in clinical radiology	Exclusion reason: Wrong study population;
Bisdas 2021	Artificial Intelligence in Medicine: A Multinational Multi-Center Survey on the Medical and Dental Students' Perception	Exclusion reason: Wrong study population;
Darcel 2023	Implementing artificial intelligence in Canadian primary care: Barriers and strategies identified through a national deliberative dialogue	Exclusion reason: Wrong study population;
Davis 2024	Adolescent, Parent, and Provider Perceptions of a Predictive Algorithm to Identify Adolescent Suicide Risk in Primary Care	Exclusion reason: Wrong study population;
Funer 2023	Responsibility and decision-making authority in using clinical decision support systems: an empirical-ethical exploration of German prospective professionals' preferences and concerns	Exclusion reason: Wrong study population;
Gillissen 2022	Medical Students' Perceptions towards Digitization and Artificial Intelligence: A Mixed-Methods Study	Exclusion reason: Wrong study population;
Hamd 2023	A closer look at the current knowledge and prospects of artificial intelligence integration in dentistry practice: A cross-sectional study	Exclusion reason: Wrong study population;
Hildebrand 2023	Study of Patient and Physician Attitudes Toward Automated Prognostic Models for Patients With Metastatic Cancer	Exclusion reason: Wrong study population;
Jeong 2023	Korean dental hygiene students' perceptions and attitudes toward artificial intelligence: An online survey	Exclusion reason: Wrong study population;
Jungmann 2021	Attitudes Toward Artificial Intelligence Among Radiologists, IT Specialists, and Industry	Exclusion reason: Wrong study population;
Labrague 2023	Student nurses' attitudes, perceived utilization, and intention to adopt artificial intelligence (AI) technology in nursing practice: A cross-sectional study	Exclusion reason: Wrong study population;
Laï 2020	Perceptions of artificial intelligence in healthcare: findings from a qualitative survey study among actors in France	Exclusion reason: Wrong study population;
Lin 2023	Revolutionising dental technologies: a qualitative study on dental technicians' perceptions of Artificial intelligence integration	Exclusion reason: Wrong study population;
Mahlknecht 2023	Supporting primary care through symptom checking artificial intelligence: a study of patient and physician attitudes in Italian general practice	Exclusion reason: Wrong study population;
Mlodzinski 2023	Assessing Barriers to Implementation of Machine Learning and Artificial Intelligence-Based Tools in Critical Care: Web-Based Survey Study	Exclusion reason: Wrong study population;
Moldt 2023	Chatbots for future docs: exploring medical students' attitudes and knowledge towards artificial intelligence and medical chatbots	Exclusion reason: Wrong study population;
Nash 2023	Perceptions of Artificial Intelligence Use in Primary Care: A Qualitative Study with Providers and Staff of Ontario Community Health Centres	Exclusion reason: Wrong study population;
Palazzi 2023	Results of the European Society of Toxicologic Pathology Survey on the Use of Artificial Intelligence in Toxicologic Pathology	Exclusion reason: Wrong study population;
Park 2021	Medical Student Perspectives on the Impact of Artificial Intelligence on the Practice of Medicine	Exclusion reason: Wrong study population;
Pelly 2023	Artificial intelligence for secondary prevention of myocardial infarction: A qualitative study of patient and health professional perspectives	Exclusion reason: Wrong study population;

PintoDosSantos 2019	Medical students' attitude towards artificial intelligence: a multicentre survey	Exclusion reason: Wrong study population;
Robleto 2024	Medical students' perceptions of an artificial intelligence (AI) assisted diagnosing program	Exclusion reason: Wrong study population;
Santos 2021	The perceptions of medical physicists towards relevance and impact of artificial intelligence	Exclusion reason: Wrong study population;
Stewart 2023	Western Australian medical students' attitudes towards artificial intelligence in healthcare	Exclusion reason: Wrong study population;
Swed 2022	Knowledge, attitude, and practice of artificial intelligence among doctors and medical students in Syria: A cross-sectional online survey	Exclusion reason: Wrong study population;
Syed 2023	Assessment of Awareness, Perceptions, and Opinions towards Artificial Intelligence among Healthcare Students in Riyadh, Saudi Arabia	Exclusion reason: Wrong study population;
Tamori 2022	Acceptance of the Use of Artificial Intelligence in Medicine Among Japan's Doctors and the Public: A Questionnaire Survey	Exclusion reason: Wrong study population;
Tangadulrat 2023	Using ChatGPT for Clinical Practice and Medical Education: Cross-Sectional Survey of Medical Students' and Physicians' Perceptions	Exclusion reason: Wrong study population;
Van der Zander 2022	Artificial intelligence in (gastrointestinal) healthcare: patients' and physicians' perspectives	Exclusion reason: Wrong study population;
Van Hoek 2019	A survey on the future of radiology among radiologists, medical students and surgeons: Students and surgeons tend to be more skeptical about artificial intelligence and radiologists may fear that other disciplines take over	Exclusion reason: Wrong study population;
Zheng 2021	Attitudes of medical workers in China toward artificial intelligence in ophthalmology: a comparative survey	Exclusion reason: Wrong study population;
Hashmi 2023	Artificial intelligence in radiology: trainees want more	Exclusion reason: Wrong study population;
Nelson 2021	Dermatologists' Perspectives on Artificial Intelligence and Augmented Intelligence - A Cross-sectional Survey	Exclusion reason: Wrong study type ;
