# Supplementary material for: Healthcare professionals’ perspectives on artificial intelligence in patient care: a systematic review of hindering and facilitating factors on different levels
Source: BMC Health Serv Res. 2025 May 1;25:633. doi: 10.1186/s12913-025-12664-2 (PMC12046968; doi:10.1186/s12913-025-12664-2)
Supplement: Supplementary file 4 — Additional file 4. Characteristics and results of included studies. [file 12913_2025_12664_MOESM4_ESM.pdf]

Additional file 4 Characteristics and results of included studies

First author	Year	Country	Study design,<br><br>Method	Sample size	Participant characteristics	Field of medicine	Facilitating factors	Hindering factors	Type of AI	AI fully implemented
Abdullah (53)	2020	Saudi Arabia	Quantitative survey,<br><br>Fuse-on questions	250	Physicians,<br>Nurses,<br>Technicians	Not specified	Time efficiency	Job replacement<br><br>Diversity	Not spec.	No
Abu-Farha (29)	2023	Jordan	Quantitative survey,<br><br>Fuse-on questions	221	Pharmacists	Pharmacy	Time efficiency<br><br>Complex cases	Communication with patient<br><br>Diversity<br><br>Dehumanization of health care	NLPM	No
Abuzaid (87)	2020	United Arab Emirates	Quantitative survey,<br><br>Fuse-on questions	153	Radiologists	Radiology	-	Lack of knowledge<br><br>Lack of education programs<br><br>Lack of responsible personal	ML/ DL*	No
Agrawal (58)	2023	USA	Quantitative survey,<br><br>Fuse-on questions	113	Radiologists	Radiology	Transparency<br><br>Sensitivity and specificity	-	ML/ DL*	No
Ahun (86)	2023	Turkey	Quantitative survey,<br><br>Mixed	167	Physicians	Emergency medicine	Non-discrimination	Responsibility	Not spec.	No
Alanzi (30)	2023	Saudi Arabia	Qualitative Focus group,<br><br>Open ended questions	54	Physicians from various medical specialties	Not specified	Time efficiency<br><br>Communication<br><br>Teamwork<br><br>Decrease time on repetitive tasks<br><br>Personalized recommendation	Lack of human touch<br><br>Diversity<br><br>Clinical error<br><br>Not up to date<br><br>Fear of dependency<br><br>AI disclosure<br><br>Security and privacy	NLPM	Yes

								Liability and accountability		
Allen (31)	2024	USA	Mixed methods,<br><br>Mixed	62	Physicians	Primary care medicine	Sensitivity and specificity	Clinical error<br><br>Patient diversity<br><br>Liability and accountability<br><br>More output<br><br>Workload<br><br>Patient doctor relationship<br><br>Overloaded by technology<br><br>Lack of reimbursement	ML<br><br>NLPM	Yes
Alsobhi (81)	2022	Saudi Arabia	Quantitative survey,<br><br>Fuse-on questions	317	Physical therapists	Physical therapy	Reduce workload	Communication with patient<br><br>Lack of human touch	Not spec.	No
Alsobhi (82)	2022	Saudi Arabia	Mixed methods,<br><br>Mixed	236	Physical therapists	Physical therapy	Reduce workload	Limitation of programming scope<br><br>Lack of human touch<br><br>Patient diversity<br><br>training costs<br><br>Development and acquisition<br><br>Lack of knowledge<br><br>diversity	Not spec.	No
Antwi (91)	2020	Africa	Qualitative survey,<br><br>Open ended questions	457	Radiologists	Radiology	-	Security and privacy<br><br>Development and acquisition<br><br>fear of job replacement	ML*	No
Asokan (75)	2023	USA	Mixed methods,<br><br>Mixed	105	Otolaryngologists	Otolaryngology	Risk assessment	Lack of knowledge<br><br>Complex cases<br><br>Reliability	ML	No

								Patient doctor relationship<br><br>Fear of dependency<br><br>Security and data privacy<br><br>Lability and accountability		
Berrami (108)	2023	Morocco	Quantitative survey,<br><br>Fuse-on questions	103	Physicians (hospital)	Not specified	-	-	Not spec.	No
Blease (77)	2019	United Kingdom	Qualitative survey,<br><br>Open ended questions	720	Physicians (GP)	Primary care medicine	Efficiency	Lack of human touch	ML	No
Blease (78)	2020	Worldwide	Quantitative, survey<br><br>Mixed	791	Psychiatrists	Psychiatry	Efficiency	Lack of human touch	ML	No
Boo (45)	2023	South Korea	Qualitative, interviews and survey<br><br>Open ended questions	15	Nurses	Not specified	Self-assurance	Usability	Not spec.	Yes
Botwe (83)	2021	Ghana	Quantitative survey<br><br>Mixed	151	Radiologists	Radiology	Workload<br><br>Clinical outcome	Clinical error	ML*	No
Buck (46)	2022	Germany	Qualitative interviews<br>Open ended questions	18	Physicians	Primary care medicine	Efficiency<br><br>Endorsement<br><br>Sensitivity and specificity	Clinical error<br><br>Patient diversity<br><br>Patient doctor relationship<br><br>privacy and security	Not spec.	No
Catalina (79)	2023	Spain	Quantitative survey,<br><br>Fuse-on questions	301	Physicians,<br>Nurses	Radiology	Improved clinical outcome<br><br>efficiency<br><br>population health	-	ML*	No
Chen (40)	2021	United Kingdom	Qualitative, interviews & focus groups	26	Radiologists	Radiology	Conferences, network<br><br>Decrease time on	Time constraints for education	ML	No

			Open ended questions				repetitive tasks<br><br>Relief workforce crisis<br><br>Clear legal framework	clinical errors		
Choi (47)	2023	South Korea	Quantitative survey,<br><br>Fuse-on questions	73	Neuroradiologists	(Neuro)Radiology	Decrease time on repetitive tasks	-	ML*	No
Coppola (59)	2021	Italy	Quantitative survey,<br><br>Fuse-on questions	1032	Radiologists	Radiology	Improved diagnostics, reduce misdiagnoses	Lack of regulatory policies	ML	No
Dlugatch (33)	2024	United Kingdom	Qualitative Interviews,<br><br>Open ended questions	13	Obstetricians, Midwives	Sexual and reproductive health	Improved diagnostics, reduce misdiagnoses<br><br>Risk assessment	Patient diversity<br><br>Research development and validation	ML*<br>RES*	No
Drogt (48)	2022	The Netherlands	Qualitative Interviews,<br><br>Open ended questions	24	Pathologists,<br>Lab technicians,<br>Computer scientists	Pathology	Complex cases<br><br>Improvement of clinical outcomes<br><br>Uniformity and evidence based<br><br>Decrease time on repetitive tasks	Implementation	Not spec.	No
Eschert (60)	2022	Germany	Quantitative survey,<br><br>Fuse-on questions	302	Dentists	Orthodontics	Uniformity and evidence based<br><br>Improved sensitivity and specificity	Liability and accountability<br><br>Security and privacy<br><br>Divestment	ML*	No
Estrada (43)	2023	USA	Quantitative survey,<br><br>Fuse-on questions	1086	Anaesthesiologists	Anaesthesiology	Timeliness of care<br><br>Efficiency<br><br>Retaining control	-	Not spec.	No
Fischer (34)	2023	The Netherlands	Qualitative interview,<br><br>Open ended questions	13	Gynaecologists	Sexual and reproductive health	Familiarity<br><br>Evidence-based AI Technology<br><br>Complex cases<br><br>Remote support	Patient diversity	ML*<br>RES*	No

Held (37)	2022	Germany	Qualitative interviews<br><br>open ended questions	24	Physicians,<br>Medical assistants,<br>Ophthalmologists	Primary care medicine	Familiarity<br><br>Clear legal framework	Integration<br><br>Lack of support<br><br>Lack of education programs<br><br>Explainability<br><br>Commercial interest	ML*	No
Hesso (39)	2023	United Kingdom, Greece, Italy, Spain, Cyprus, Serbia	Mixed methods,<br><br>Mixed	122	Physicians from various medical specialties	Oncology	Evidence-based technology<br><br>Decision support and not replacement<br><br>User friendliness<br><br>Education training<br><br>Time to trial AI	Implementation<br><br>Explainability<br><br>Time constraint<br><br>Age	ML	No
Hindocha (52)	2023	United Kingdom	Quantitative survey<br><br>Mixed	51	Consultants	Oncology	Efficiency<br><br>More time / improved focus on patient	Lack of regulatory policies<br><br>Lack of education programs	ML*	No
Ho (61)	2022	Australia	Quantitative survey,<br><br>Mixed	133	Optometrists	Ophthalmology	Sensitivity and specificity<br><br>Access to health care<br><br>Second opinion	-	ML	No
Högberg (62)	2023	Sweden	Quantitative survey,<br><br>Mixed	47	(Breast) radiologists	Radiology	Sensitivity and specificity<br><br>Consistency of care<br><br>Second opinion	Sensitivity / specificity<br><br>Fear of dependency<br><br>Accountability and liability<br><br>Increased workload<br><br>Health inequalities<br><br>Patient diversity	ML*	No
Holzner (49)	2022	Germany	Qualitative interviews<br><br>open ended questions	12	Physicians from various medical specialties	Not specified	Sensitivity and specificity<br><br>Time efficiency<br><br>Decrease time on repetitive tasks<br><br>Patient safety	Patient doctor relationship<br><br>Security and privacy<br><br>Clinical error	Not spec.	No

							Process optimization<br><br>Consistency of care	Lack of human touch<br><br>Inadequacy in certain context		
Kamal (88)	2023	Sudan	Qualitative interviews<br><br>Fuse on	128	Surgeons	Orthopaedics	-	Knowledge of AI	Not spec.	No
Khafaji (63)	2022	Saudi Arabia	Quantitative survey,<br><br>Fuse-on questions	154	Residents	Radiology	Sensitivity and specificity<br><br>Consistency<br><br>Time efficiency	-	ML	No
Horsfall (57)	2021	United Kingdom, India	Mixed method,<br><br>mixed	133	Neurosurgeons, Anaesthetists, Nurses, Operating room practitioners	Neurosurgery	Process optimization<br><br>Teamwork / coordination<br><br>Real time alert	-	Not spec.	No
Leenhardt (41)	2021	Multiple 20 Countries	Quantitative survey,<br><br>Fuse-on questions	380	Gastroenterologists, Nurses, Technicians	Radiology (Capsule endoscopy)	Efficiency<br><br>No fear of job loss	Responsibility	ML*	No
Lin (64)	2023	China	Quantitative survey,<br><br>Fuse-on questions	480	Orthodontists, Medical students	Orthodontics	Efficiency<br><br>Sensitivity and specificity	-	ML*	No
Maassen (74)	2021	Germany	Quantitative survey,<br><br>Fuse-on questions	121	Physicians from various medical specialties	Not specified	Complex cases<br><br>Process optimization	Job requirements<br><br>Fear of dependency<br><br>Evidence-based technology	Not spec.	No
Martinho (44)	2021	The Netherlands, Portugal, USA	Quantitative survey,<br><br>Mixed	77	Physicians from various medical specialties	Not specified	Not replacement<br><br>More time, focus on patient<br><br>Decision support	Privacy and security<br><br>Lack of regulatory policies<br><br>Patient doctor relationship	Not spec.	No
Martiniussen (65)	2023	Norway	Quantitative survey,<br><br>Fuse-on questions	60	Radiologists	Radiology	Sensitivity and specificity<br><br>Efficiency	-	ML*	No
Massey (66)	2023	USA	Mixed- Mixed methods,<br><br>Mixed	139	Otolaryngologists, Rhinologists	Radiology	Standardized reporting<br><br>Scientific objectivity<br><br>Sensitivity and specificity	Fear of dependency<br><br>Reliability<br><br>Insurance companies	ML*	No

								Lack of knowledge		
Nadarzynski (32)	2023	United Kingdom	Mixed methods,<br><br>Mixed	174	Physicians,<br>Nurses	Sexual and reproductive health	Access to healthcare<br><br>Personalized recommendation<br><br>Communication with patients<br><br>Patient safety	Lack of human touch<br><br>Health inequality<br><br>Lack of knowledge<br><br>Patient diversity<br><br>Limitation of programming scope<br><br>Workload	NLPM*	No
Ng (50)	2022	Singapore	Qualitative focus groups<br><br>Open ended questions	22	Radiologists	Radiology	Efficiency<br><br>Improvement of clinical outcomes<br><br>Reduce time on repetitive tasks<br><br>Sensitivity and specificity	Clinical error<br><br>Fear of replacement<br><br>Lack of knowledge<br><br>Patient diversity	Not spec.	No
Oh (92)	2019	Korea	Quantitative survey,<br><br>Fuse-on questions	669	Medical students, Physicians	Not specified	-	Conflict of opinion	Not spec.	No
Orlova (55)	2023	Russia	Quantitative survey,<br><br>Fuse-on questions	301	Physicians from various medical specialties	Not specified	Handling of big data	-	Not spec.	No
O'Shaughnessey (109)	2023	Australia	Mixed methods,<br><br>Mixed	105	Radiologists	Radiology	-	-	ML*	No
Patrzyk (85)	2022	Poland	Quantitative survey,<br><br>Fuse-on questions	90	Dermatologists	Dermatology, Venereology	-	Clinical error	Not spec.	No
Pecqueux (67)	2022	Germany	Quantitative survey,<br><br>Fuse-on questions	147	Surgeons	Surgery<br>(various disciplines)	Sensitivity and specificity	Accountability	Not spec.	No
Pedro (51)	2023	Portugal	Quantitative survey,<br><br>Fuse-on questions	1013	Physicians from various medical specialties	Not specified	Task delegation<br><br>Time efficiency	Lack of human touch	Not spec.	No

Perrier (68)	2022	France	Quantitative survey,<br><br>Fuse-on questions	165	Pediatricians	Paediatricians	Access to health care<br><br>Sensitivity and specificity	Lack of knowledge	Not spec.	No
Rainey (93)	2022	United Kingdom	Quantitative survey,<br><br>Mixed	86	Radiologists	Radiology	-	Reliability	ML*	No
Rho (69)	2022	Korea	Mixed method,<br><br>Mixed	86	Urologists	Oncology	Sensitivity and specificity<br><br>Efficiency	Treatment methods and digital systems	ML*	Yes
Romero-Brufau (35)	2020	USA	Quantitative survey,<br><br>Fuse-on questions	81	Physicians,<br>Nurses	Not specified	Teamwork	Patient diversity	RES	Yes
Ryan (110)	2021	Ireland	Mixed methods,<br><br>Mixed	395	Radiologists	Radiology	-	-	ML	No
Sahin (56)	2023	Turkey	Quantitative survey,<br><br>Mixed	156	Oncologists	Oncology	Handling big data	Lack of human touch	Not spec.	No
Samhammer (111)	2022	Germany	Qualitative interviews,<br><br>Open ended questions	14	Nephrologists	Nephrology	-	-	ML (DSS)	Yes
Sangers (38)	2023	Netherlands	Qualitative focus groups,<br><br>Open ended questions	33	Dermatologists,<br>Physicians	Dermatology	Familiarity<br><br>Sensitivity and specificity<br><br>Time efficiency<br><br>Cost efficiency<br><br>Education tool<br><br>Endorsement<br><br>Clear legal framework<br><br>Evidence-based technology	Bias in training data<br><br>Unequal health quality<br><br>Fear of job replacement<br><br>Workload<br><br>Commercial interest<br><br>Security and data privacy<br><br>Usability	Not spec.	No
Sarwar (112)	2019	Canada	Quantitative survey,<br><br>Fuse-on questions	487	Pathologists	Pathology	-	-	Not spec.	No
Savoia (70)	2024	Germany	Quantitative survey,	404	Nephrologists	Haemodialysis	Sensitivity and specificity	-	Not spec.	No

			Fuse-on questions				Patient safety			
Scanzera (71)	2022	USA	Quantitative survey,<br><br>Mixed	400	Optometrists	Ophthalmology	Sensitivity and specificity	Overreliance of technology	Not spec.	No
Shelmerdine (42)	2022	United Kingdom	Quantitative survey,<br><br>Mixed	240	Radiologists	Radiology	No fear of job loss<br><br>Real time alert of hazards and complications	Evidence-based technology	ML	No
Shiang (84)	2022	USA	Mixed methods,<br><br>Mixed	15	Residents	Radiology	Process optimization	Clinical errors	ML (DSS)	Yes
Shin (72)	2023	USA	Quantitative survey,<br><br>Fuse-on questions	120	Radiologists	Radiology	Sensitivity and specificity	-	ML*	No
Shinners (90)	2023	Australia	Quantitative survey,<br><br>Fuse-on questions	176	Health employees	Not specified	-	Lack of education program	Not spec.	No
Staartjes (113)	2020	Switzerland	Quantitative survey,<br><br>Fuse-on questions	362	Neurosurgeons	Neurosurgery	-	-	ML	No
Syed (76)	2024	Saudi Arabia	Quantitative survey,<br><br>Fuse-on questions	258	Pharmacists	Pharmacy	Access to health care<br><br>Decision support	Lack of knowledge	Not spec.	No
Tokgöz (80)	2023	Germany	Qualitative interviews,<br><br>Open ended questions	14	Physicians	Not specified	Efficiency<br><br>Quality of care	Lack of knowledge<br><br>Liability	ML (DSS)	No
Van Cauwenberge (54)	2022	Belgium	Mixed methods,<br><br>Mixed	24	Physicians	Not specified	Patient safety<br><br>Time efficiency	-	ML (DSS)	Yes
Valikodath (89)	2021	USA	Quantitative survey,<br><br>Fuse-on questions	80	Ophthalmologists	Ophthalmology	-	Lack of knwoledge	Not spec.	No
Vijayakumar (36)	2023	Singapore	Qualitative focus groups,	12	Physicians, Medical students	Not specified	Retaining control<br><br>Health improvement	Lack of transparency	RES	Yes

			Open ended questions				Side effects<br><br>Collaboration			
Wadhwa (73)	2020	USA	Quantitative survey,<br><br>Fuse-on questions	123	Gastroenterologists	Gastroenterology	Sensitivity and specificity	Implementation	ML*	Yes
Wewetzer (94)	2023	Germany	Quantitative survey,<br><br>Fuse-on questions	209	Physicians	Not specified	-	Costs	ML*	No

Not spec.= not specified, ML=machine learning, NLPM=natural language processing model, RES=rule-based expert systems, DSS= decision support systems.
